# Billing Opinion Reliability Score 100: A Structured Framework for Billing and Medicolegal Expert Reliability

**DOI:** 10.7759/cureus.112462

**Published:** 2026-07-11

**Authors:** Andrew M Klapper, Anthony N Dardano, Michael S Risin, Karla Maita, Monali Mahedia

**Affiliations:** 1 Plastic and Reconstructive Surgery, Delray Medical Center, Delray Beach, USA

**Keywords:** benchmark fitness, bors100, coding, expert reliability, independent dispute resolution, medical billing, medicolegal opinion, no surprises act, reimbursement, structured assessment

## Abstract

Medical billing, coding, reimbursement, No Surprises Act Independent Dispute Resolution (NSA/IDR), and medicolegal disputes frequently depend on expert opinions that vary substantially in transparency, evidentiary support, methodological discipline, and case-specific reasoning. Existing legal and administrative processes may assess expert reliability, but they do not provide a uniform rubric for evaluating the internal methodological quality of billing and reimbursement expert opinions across dispute settings. Billing Opinion Reliability Score 100 (BORS100) was developed as a proposed conceptual structured reliability assessment framework for expert opinions involving medical billing, coding, reimbursement, NSA/IDR, and related medicolegal questions. Domains were identified through a three-step process: structured review of published expert testimony standards, structured professional judgment literature, and psychometric instrument development principles; extraction of recurring reliability limitation categories; and iterative consolidation into seven scoring domains. The framework is intended to generate a domain-level reliability profile supported by a narrative explanation. It is not a validated psychometric instrument, a legal admissibility test, or a substitute for adjudicator judgment. BORS100 contains seven scoring domains: opinion scope and qualification fit (15 points); source and documentation support (15 points); methodology and reproducibility (20 points); benchmark literacy and fitness-for-purpose (15 points); case-specific clinical or procedural linkage (15 points); transparency and auditability (10 points); and bias and independence disclosure (10 points). Proposed interpretation bands range from highly structured and well-documented opinion profiles (85-100) to profiles with severe evaluability limitations (below 40). These bands are proposed as descriptive categories for pilot use and require empirical calibration before adoption as institutional thresholds, comparative rankings, or decision rules. BORS100 offers a structured vocabulary for assessing whether a billing or reimbursement expert opinion discloses the scope, data, method, benchmark logic, case-specific linkage, calculations, and conflicts necessary for independent review. Its current value is organizational rather than determinative: it makes visible the records reviewed, methods used, benchmark fitness analysis, case-specific linkage, audit trail, and disclosure practices supporting the opinion. Until empirical validation is completed, BORS100 should be used as a structured review aid and pilot framework, not as a binding standard, objective measurement instrument, legal admissibility test, or payment determination formula.

## Introduction

Expert opinions are used in medical billing, coding, reimbursement, No Surprises Act (NSA) Independent Dispute Resolution (IDR), lien disputes, workers’ compensation, payer-provider disputes, personal injury litigation, and related medicolegal settings [1-5]. These opinions may address coding accuracy, documentation sufficiency, billed-charge reasonableness, payment reasonableness, benchmark interpretation, payer-policy compliance, or the relationship between clinical complexity and reimbursement.

In this context, billed charges refer to the amount listed by the provider, allowed amounts refer to the amount a payer determines is payable, paid amounts refer to actual disbursement after patient cost-sharing, and usual and customary charge references generally describe charge distributions in a defined market. The qualifying payment amount (QPA) is an NSA reference amount generally based on plan-specific median in-network contracted rates for a given item or service. Benchmark fitness refers to whether a cited benchmark measures the same construct that the expert is using to support.

Billing and reimbursement opinions may draw on expertise from certified coders, medical auditors, reimbursement analysts, physicians, health economists, compliance professionals, and payer-policy specialists. Each background may contribute relevant knowledge within an appropriately bounded scope. The structural challenge across these contexts is that opinion scope, qualification basis, analytical method, and case-specific application vary substantially and are not consistently disclosed in ways that permit independent review. This variation limits the ability of adjudicators, parties, and reviewers to distinguish documentation-supported reasoning from conclusory assertions.

Billing Opinion Reliability Score 100 (BORS100) is proposed as a structured reliability assessment framework for organizing a review of these opinions. It is intended to make visible the elements that permit an opinion to be evaluated: the exact opinion offered, the qualification basis for that opinion, the records and data reviewed, the benchmarks selected or rejected, the analytical steps used, the clinical or procedural facts considered when relevant, the calculations performed, the limitations acknowledged, and the disclosures needed to assess independence. BORS100 does not determine truth, legal admissibility, payment obligation, or adjudicative outcome. It is a decision-support aid for structured analysis, and its score should not be interpreted as an objective measure of testimony quality absent empirical validation.

The authors are not aware of a published framework that specifically proposes a structured, domain-scored reliability rubric for billing and reimbursement expert opinions. This absence creates a practical problem: opinion quality is often assessed informally, inconsistently, and without a shared vocabulary for distinguishing disclosed reasoning from conclusory assertion. BORS100 is proposed to fill that gap as a conceptual framework requiring future validation, not as a finished measurement instrument.

The purpose of this proposal is to define a shared review vocabulary and domain structure. The framework is intended to improve transparency in the review process, not to transform expert opinion review into a numerical adjudication exercise.

As a practical example, a low-evaluability billing opinion might state that a surgical charge is unreasonable because it exceeds Medicare payment rates, without explaining why Medicare is a fit benchmark for the dispute, whether the operative record was reviewed, or how the reduction was calculated. A higher-evaluability opinion would define the exact question being answered, list the records and benchmarks reviewed, explain what each benchmark measures and excludes, link the analysis to the specific clinical or billing facts, disclose assumptions, and show the calculation path.

At this stage, BORS100 is best understood as a conceptual framework and structured checklist with a preliminary pilot-use scoring component. It is not a validated rating system, psychometric instrument, legal admissibility standard, payment formula, or substitute for adjudicator judgment.

Relationship to existing reliability standards

Federal Rule of Evidence 702, Daubert, and Kumho Tire address expert reliability in the context of federal court proceedings in the United States [1-4]. The 2023 amendment to Federal Rule of Evidence 702 clarified that the proponent of expert testimony bears the burden of demonstrating admissibility by a preponderance of the evidence and that an expert may not simply assert that an opinion is the product of reliable principles and methods: the opinion must, in fact, reflect reliable application to the specific facts. This amendment strengthened the gatekeeping role of trial courts and signaled heightened scrutiny of conclusory expert opinions that lack demonstrated methodological transparency.

BORS100 draws on the general principles that those authorities articulate, i.e., defined scope, sufficient facts or data, reliable methods, and reliable application to facts, but does not present itself as a legal admissibility test, a substitute for judicial gatekeeping, or an implementation of those authorities. Many billing and reimbursement opinions are used outside courtroom settings where formal evidentiary rules do not apply: IDR, internal appeals, lien resolution, payer-provider negotiations, and administrative review all depend on expert reasoning without formal gatekeeping. BORS100 addresses the broader need for a transparent, structured review in all those settings, not only in litigation. The framework is not a legal test, and any reference to it in a legal, arbitral, or administrative proceeding should be accompanied by its limitations and should not be presented as an independent rule of decision.

## Technical report

Rationale for a structured expert reliability framework

Unstructured expert review is vulnerable to overreliance on credentials, institutional affiliation, familiar benchmarks, polished formatting, or conclusory experience statements. A written opinion that cites Medicare rates, FAIR Health values, qualifying payment amounts, payer fee schedules, or usual-and-customary references may appear technically grounded even when it does not explain why those sources are fit for the specific dispute, what they measure, or what they exclude. Conversely, a well-reasoned opinion may be difficult to evaluate if its assumptions, exclusions, and calculations are not organized transparently.

Existing structured review frameworks in medical education and evidence synthesis, such as GRADE (Grading of Recommendations, Assessment, Development and Evaluation) for clinical evidence quality [6], ROBIS (Risk of Bias in Systematic Reviews) for systematic review bias [7], and Institute of Medicine criteria for clinical practice guideline development [8], were developed for evaluating clinical evidence quality, systematic review bias, and clinical practice guideline development, respectively. None addresses the specific reliability dimensions of billing and reimbursement expert opinions: benchmark fitness-for-purpose analysis, qualification-to-opinion-lane fit, and the interaction between clinical complexity and charge reasonableness reasoning. BORS100 does not replace or compete with those frameworks; it addresses a domain that those frameworks were not designed to cover. Like them, BORS100 applies the same organizing principle, i.e., explicit criteria, disclosed reasoning, and reproducible application, to expert opinion review in billing and reimbursement disputes.

An opinion is considered evaluable from disclosed materials when a qualified reviewer, relying only on the written opinion, curriculum vitae, records-reviewed list, source documents, workpapers, calculations, and disclosure statements, can identify the opinion’s scope, follow the analytical steps, verify the data sources, assess the benchmark fitness, and check the calculations without requiring access to the original witness. An opinion that cannot disclose its scope, data, method, benchmark logic, clinical linkage, calculations, and conflicts is not meaningfully evaluable regardless of the expertise or credentials of its author. This principle, evaluability from disclosed materials, is the organizing standard of the framework.

The framework is deliberately symmetrical in design. BORS100 applies to opinions offered by physicians, payers, plaintiffs, defendants, providers, lienholders, reimbursement consultants, coding experts, or health economists without preference for the party offering the opinion. A provider-side opinion defending a charge scores lower if it lacks a disclosed method, benchmark fitness analysis, or auditability, just as a payer-side opinion challenging a charge scores lower for the same documented limitations. The framework evaluates the documented reliability of the opinion process, not the substantive position of the party offering it.

The framework does not privilege credentials over method. An experienced billing analyst who provides a stepwise written analysis, discloses the records reviewed, identifies the coding standards applied, and traces the reasoning from evidence to conclusion will score higher on Domain 3 than a credentialed clinician who offers only a conclusory opinion. The framework evaluates demonstrated and documented reasoning, not professional background or party affiliation.

Two methodologically adequate opinions may reach different conclusions because they apply different benchmark hierarchies, different clinical relevance assumptions, or different interpretations of the applicable standard. BORS100 does not resolve that disagreement; it confirms only that both opinions are evaluable. The disagreement is then a legitimate professional dispute whose resolution belongs to the adjudicator. A low BORS100 score identifies documentation and methodological gaps that limit the evaluability of the opinion; it does not prove that the conclusion is incorrect or that the witness acted in bad faith.

Conceptual basis of BORS100

BORS100 rests on four conceptual principles drawn from general standards for expert testimony reliability, psychometric validation, and structured professional judgment [1-4,9-13].

Defined Opinion Scope

Coding accuracy, payment reasonableness, billed charge reasonableness, medical necessity, operative complexity, payer-policy compliance, and legal damages reasonableness are related but distinct questions. Each requires different expertise, different evidence, and potentially different legal or regulatory standards. An opinion that blends these categories without acknowledging their distinctions cannot be evaluated against the appropriate standard for any of them.

Qualification-to-Opinion Fit

General billing experience may support some opinions but not others, particularly when clinical complexity, medical necessity, or health-economic methodology is at issue. The relevant question is not whether the witness has credentials but whether those credentials match the exact opinion offered. An emergency physician with documented experience in emergency facility billing who explicitly links that experience to the specific coding question demonstrates a high qualification fit. A general billing consultant whose documented experience is in payer-side claims management, opining on emergency surgical complexity without relevant clinical training, demonstrates a low qualification fit. An opinion that exceeds the qualifying expertise lane introduces conclusions that cannot be evaluated by the stated expertise basis.

Documentation-Supported, Reproducible Method

Experience can explain familiarity with a field, but it does not by itself disclose the steps used to reach a conclusion. A reliable opinion should identify records reviewed, data sources, assumptions, exclusions, calculations, benchmark selection rationale, alternative benchmarks considered, and limitations. This requirement does not mandate a particular method; it requires that whatever method was used be visible and evaluable. The presence of a comparison table or percentage calculation does not satisfy this requirement if the derivation of the input values is not explained; the methodology is the derivation process, not its output.

Case-Specific Application

In clinically complex procedural matters, the CPT code or billing form is not a substitute for the operative report, clinical record, emergency context, modifier rationale, or relevant procedural facts. An opinion that does not engage with the specific clinical or procedural facts at issue in the dispute has not reviewed the subject of the opinion. Where an opinion addresses a purely administrative coding, contract interpretation, or nonclinical question, the clinical linkage requirement should be scaled to the clinical relevance of that question.

These principles are compatible with legal reliability concepts but are not presented as legal doctrine and are not specific to any jurisdiction, court system, or administrative process.

Methodology and framework development

Given that BORS100 is a proposed conceptual framework rather than an empirical study, this Technical Report is organized around rationale, conceptual basis, framework development, scoring domain description, use cases and boundaries, legal and administrative relevance, limitations, and future validation pathways. The abstract uses standard IMRAD category labels for cross-database indexing.

**Figure 1:**
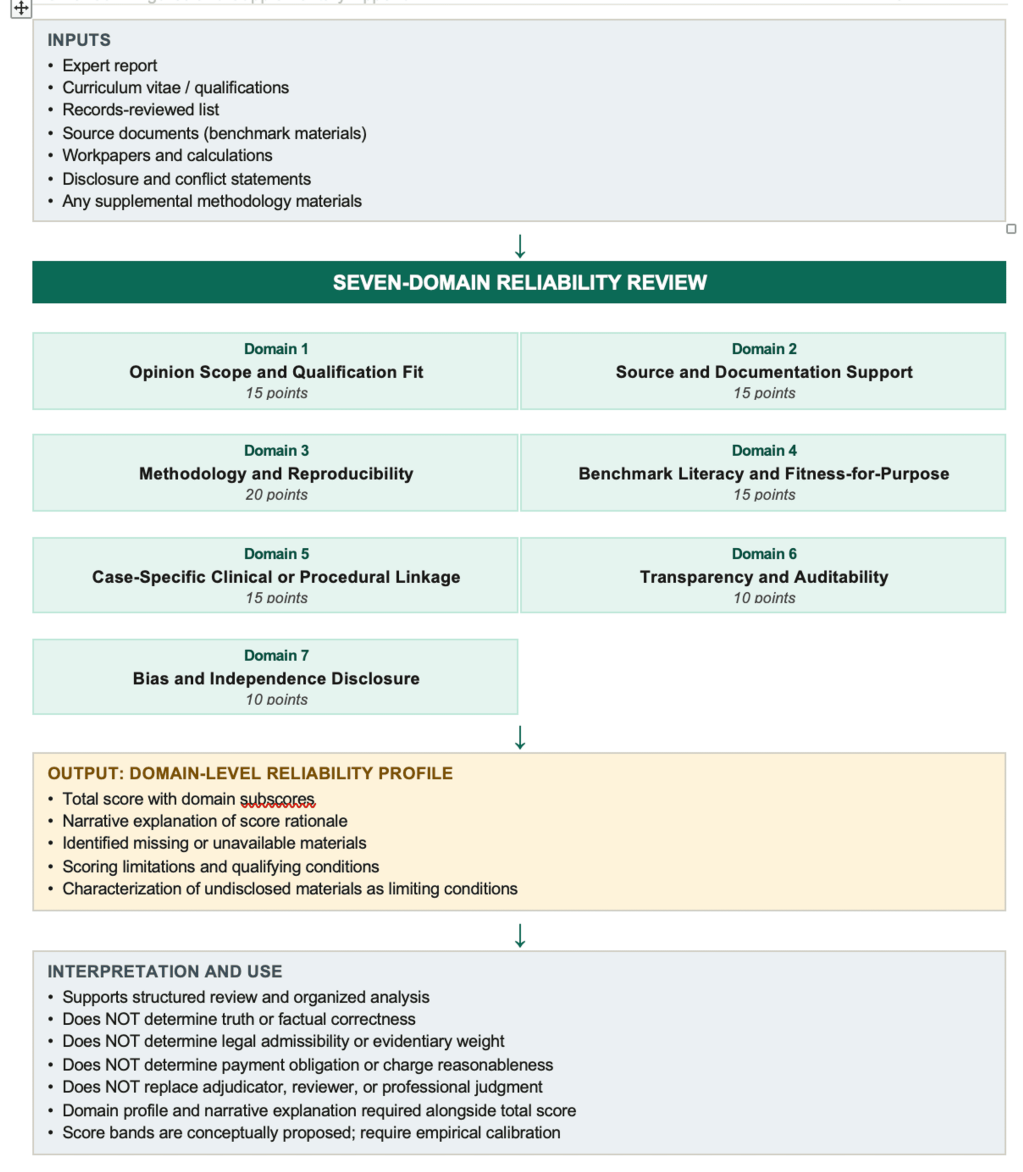
BORS100 conceptual model. Inputs from the expert report, curriculum vitae, records-reviewed list, source documents, benchmark materials, workpapers, calculations, and disclosure statements are organized across seven reliability domains. The resulting output is a domain-level reliability profile supported by narrative explanation, including domain subscores, identified missing materials, and scoring limitations. The profile supports structured review but does not determine truth, legal admissibility, payment obligation, or adjudicative outcome. Adjudicator, reviewer, and professional judgment remain primary. BORS100: Billing Opinion Reliability Score 100

BORS100 was developed through a three-step domain identification process. The overall BORS100 review structure is summarized in Figure 1, which shows how disclosed opinion materials are organized across seven reliability domains to generate a domain-level reliability profile with narrative explanation and identified limitations.

First, a targeted narrative review was conducted across published analyses of expert testimony standards under Federal Rule of Evidence 702 and Daubert, structured professional judgment frameworks from forensic and actuarial contexts [12,13], psychometric instrument development principles [9-11], and published materials addressing billing, coding, reimbursement, and benchmark interpretation. The review was conducted as a targeted narrative review rather than a systematic review. Sources were identified through PubMed, Google Scholar, federal rules and regulatory materials, legal reference searches, and reference chain review. Search concepts included expert testimony reliability, Federal Rule of Evidence 702, Daubert, structured professional judgment, psychometric validation, medical billing expert opinion, reimbursement benchmarks, qualifying payment amount, usual and customary charges, FAIR Health, Medicare Physician Fee Schedule, independent dispute resolution, and charge reasonableness. Materials were included when they addressed expert opinion reliability, structured review methods, psychometric or scoring framework development, billing or reimbursement benchmark interpretation, or administrative dispute review processes. Materials were excluded when they addressed clinical evidence grading without transferable framework development principles or reimbursement policy without relevance to expert opinion reliability. The review was intended to identify candidate reliability domains and recurring failure modes, not to function as a systematic review, meta-analysis, or evidence synthesis. Second, recurring reliability limitation categories were extracted from those sources and organized by functional type: scope limitation, qualification mismatch, methodological opacity, benchmark misapplication, clinical record disengagement, auditability failure, and independence non-disclosure. Third, those categories were consolidated into seven scoring domains through iterative reconciliation to minimize conceptual overlap while preserving distinct reliability dimensions.

The resulting domains were mapped to recurring source concepts as follows: scope definition and qualification fit were derived from expert testimony reliability principles; source support, methodology, reproducibility, and auditability were derived from reliability, psychometric, and structured review principles; benchmark fitness was derived from reimbursement and health policy benchmark literature; case-specific linkage was derived from requirements that opinions apply methods to the specific facts reviewed; and bias disclosure was derived from expert testimony and conflict disclosure principles.

No primary empirical data were collected. The resulting domain structure is the authors’ proposed synthesis and has not been independently validated by external consensus panels or expert surveys; such validation is identified as a priority in the future validation pathway.

The framework was developed by the author group and has not yet undergone formal external consensus review by payer, provider, legal, coding, arbitration, or health economic stakeholders. To reduce directional bias during drafting, the domains were written to apply symmetrically to all party positions and expert backgrounds, and misuse safeguards were added throughout the manuscript and worksheet. However, symmetry in drafting is not a substitute for independent stakeholder validation. External multidisciplinary review remains necessary before institutional or high-stakes use.

The point allocation gives the greatest weight to methodology and reproducibility (20 points), and equal secondary weight to opinion scope and qualification fit, source and documentation support, benchmark literacy and fitness-for-purpose, and case-specific clinical or procedural linkage (15 points each). Transparency and auditability (10 points) and bias and independence disclosure (10 points) receive lower but non-trivial weight.

The rationale for differential weighting is as follows: methodology and reproducibility (Domain 3) receives the highest weight because it is the domain least amenable to post hoc supplementation. Missing records can be disclosed after the fact; undisclosed conflicts can be reported, but a method that was not applied cannot be reconstructed. This asymmetry justifies the higher weight without requiring that methodology be considered more important than other domains in every dispute context. Scope and qualification fit, source support, benchmark fitness, and case-specific linkage receive equal secondary weight because each represents a distinct evaluability dimension of comparable importance across dispute types. Transparency and disclosure domains receive lower but non-trivial weight because they condition the evaluability of the other domains without fully substituting for them. These weights should be treated as a conceptual starting point for pilot testing, not as established empirical findings. Future validation should examine whether the current weights produce meaningfully different rankings compared with equal-weight alternatives.

Equal weighting was considered as an alternative approach because all seven domains contribute to evaluability. The preliminary differential weighting was retained because methodology and reproducibility were judged to be less correctable after the fact than disclosure, source listing, or other elements that may be supplemented. This choice remains conceptual rather than empirical, and future validation should compare the current weighting structure with equal weight and empirically derived alternatives.

The additive 100-point structure implicitly assumes that domain scores can be meaningfully combined. In the current framework, the total score should be understood as an approximate summary of how the opinion performs across seven review categories, useful for structuring reviews and identifying missing elements, rather than as a scale with demonstrated interval properties. The 100-point structure was selected because it communicates domain proportionality intuitively and facilitates descriptive comparison; it was not selected because 100 is the required scale for the construct. Analyses treating the total score as a continuous variable with arithmetic meaning should be approached with caution until the measurement properties of the scale are established.

BORS100 scoring domains

Table 1 presents the seven scoring domains with maximum point allocations, what each domain measures, and examples of high- and low-reliability features.

**Table 1:** BORS100 domains and point allocation. BORS100: Billing Opinion Reliability Score 100

Domain	Maximum	What it measures	High-reliability features	Low-reliability features
Opinion scope and qualification fit	15	Whether the opinion category is defined and expert qualifications match that exact category	Explicit scope statement; limits stated; qualification-to-opinion linkage demonstrated; opinion categories distinguished	Blended scope; credential used outside the demonstrated lane; clinical complexity opinion without a clinical basis
Source and documentation support	15	Whether the opinion identifies and uses records, data sources, and documentation sufficient for independent review	Records reviewed list with dates; source versions disclosed; data provenance; exclusions disclosed and explained	Unlisted records; missing source versions; unexplained omissions; record selection appearing limited to favorable materials without explanation
Methodology and reproducibility	20	Whether the reasoning process is visible and could be followed by another qualified reviewer using the same disclosed inputs	Stepwise method; assumptions stated; calculations disclosed; alternative analyses; derivation process visible	Experience-only reasoning without steps; black-box conclusion; no workpapers; benchmark derivation unexplained
Benchmark literacy and fitness-for-purpose	15	Whether benchmarks are interpreted according to what they measure and exclude, and why they apply to this specific dispute	Benchmark limits and selection rationale explained; alternative benchmarks discussed; structural characteristics addressed; both directions of substitution considered	Benchmark recitation without explanation; benchmark substitution without justification in either direction; no alternative benchmarks discussed
Case-specific clinical or procedural linkage	15	Whether clinical or procedural facts are considered when relevant; scaled to opinion type; administrative opinions exempt from clinical record requirement	Operative record reviewed when relevant; acuity, modifiers, site, and complexity addressed; deferral stated where appropriate; administrative opinions appropriately scoped	CPT descriptor substituted for clinical record in complex cases; no complexity linkage when directly relevant; scaling not applied
Transparency and auditability	10	Whether another reviewer can audit the analysis from the disclosed materials without access to the original witness	Raw values disclosed; step-by-step calculations traceable; source versions identified; missing data disclosure; complete data path	No traceable calculation path; benchmark versions undisclosed; unreproducible arithmetic; incomplete data path
Bias and independence disclosure	10	Whether financial, retention, and role-based incentives are disclosed; applies symmetrically to all party positions and expert backgrounds	Retention history; income source distribution; party mix; prior testimony when applicable; relevant financial relationships, from any retained party	No disclosure of any kind; one-sided history undisclosed; incomplete conflict statement, whether provider-side, payer-side, defense-side, or lien-side
Total	100			

Some conceptual overlap among domains is expected because reliable opinions require integration of scope, data, method, benchmark interpretation, and case-specific application. To reduce double-counting and improve inter-rater consistency, each domain should be scored only for the feature it directly measures, and raters should document the specific basis for each score from the disclosed materials.

Points should be awarded only for features visible in the disclosed record: the written opinion, curriculum vitae, records reviewed list, source documents, workpapers, calculations, disclosure statements, and any supplemental methodology materials. Assertions that a factor was considered should receive credit only when the consideration is demonstrable in the disclosed record. When relevant materials are unavailable, raters should identify the absence as a scoring limitation and characterize scores accordingly.

If an expert reports that an analysis was performed but does not disclose the supporting materials, calculations, assumptions, or data path, the reviewer should not score the undisclosed analysis as fully demonstrated. The appropriate response is to identify the item as a limiting condition and, when feasible, request supplementation. BORS100 evaluates disclosed evaluability, not undisclosed effort.

BORS100 is designed for use by qualified reviewers with sufficient domain knowledge to evaluate the disclosed materials against the scoring criteria. Reviewers should have familiarity with medical billing and reimbursement principles, relevant benchmarks, and documentation standards applicable to the opinion type being reviewed. A standardized training process, including scoring manual review, worked examples, and calibration exercises, is recommended before formal use.

The framework also functions as a preparation and quality improvement tool. A physician expert preparing a charge reasonableness opinion can use the domain structure to identify which elements, explicit scope statement, records reviewed list, benchmark fitness analysis, calculation trail, and conflict disclosure, should be documented before the opinion is finalized. A non-physician reimbursement expert can use the same structure to confirm that the opinion remains within the qualification lane and that every numerical conclusion has a traceable source. Using the domain structure as a preparation checklist does not depend on validation of the numerical score, provided the framework is accurately described as a checklist and not as a validated scoring instrument.

Prior use of BORS100 during report preparation may improve documentation and therefore increase subsequent BORS100 scores. That effect is not necessarily a flaw when the framework is used as a quality improvement checklist, because improved disclosure and auditability are intended outputs. However, when BORS100 is used to compare independently prepared opinions, prior self-review or checklist use should be disclosed because it may affect score interpretation and reduce the framework’s discriminative value.

Domain 1: Opinion Scope and Qualification Fit (15 Points)

This domain evaluates whether the opinion category is explicitly defined and whether the stated qualifications demonstrably match that category. High-reliability features include an explicit scope statement identifying what is and is not being opined on, a visible linkage between the credential basis and the specific opinion offered, and an acknowledgment of the boundary between different opinion categories. Low-reliability features include blended or undefined scope, use of general billing credentials for opinions requiring clinical or health economic qualification, and extension into opinion categories not supported by the stated expertise.

Domain 2: Source and Documentation Support (15 Points)

This domain evaluates whether the opinion identifies and uses records, data sources, and documentation sufficient for independent review. High-reliability features include a records reviewed list with dates, source identification with version and date, provenance disclosure, and explicit identification of records excluded and the basis for exclusion. Low-reliability features include unlisted records, missing source versions, unexplained omissions, failure to identify the specific data products used, and a record selection process that appears limited to materials favorable to the opinion’s conclusion without explanation of why unfavorable or neutral records were excluded or not reviewed.

Domain 3: Methodology and Reproducibility (20 Points)

This domain evaluates whether the reasoning process is visible and could be followed by another qualified reviewer applying the same disclosed inputs. High-reliability features include a stepwise written method, stated assumptions, disclosed calculations, identified limitations, and alternative analyses or sensitivity testing. Low-reliability features include experience-only reasoning without accompanying methodological steps, black-box conclusions, absence of workpapers or calculations, and unexplained jumps from data to conclusion.

The domain does not penalize non-credentialed reviewers or penalize experience per se. An experienced billing analyst who provides a stepwise written analysis, identifies the records reviewed, applies stated coding standards, and traces the reasoning from evidence to conclusion will score higher on this domain than a credentialed physician who provides only a conclusory opinion. A comparison table or percentage calculation may be present without satisfying this domain if the derivation of the input values is not explained; the methodology is the derivation process, not its output.

Domain 4: Benchmark Literacy and Fitness-for-Purpose (15 Points)

This domain evaluates whether benchmarks are interpreted according to what they actually measure and exclude, and whether their selection is justified for the specific dispute. Benchmark constructs appearing in billing and reimbursement disputes include the following, each measuring a distinct dimension of the payment ecosystem [5,14-18].

The Medicare Physician Fee Schedule reflects resource-based relative values converted to payment amounts under a federal fee schedule model that excludes out-of-network private market charges and does not represent billed charges or negotiated commercial rates [18]. FAIR Health charge benchmarks represent the distribution of submitted charges in defined geographic markets at specified percentiles; FAIR Health-allowed benchmarks represent estimated reimbursement rates [16,17]. QPAs under 45 C.F.R. § 149.140 are calculated from in-network median contracted rates on a plan-specific and service-specific basis and are used in the NSA/IDR context as a starting point for arbitration, not as a universal charge reasonableness standard; two payers submitting to the same IDR for the same service in the same market may have different QPAs. Usual, customary, and reasonable charges are typically derived from commercial charge databases, such as FAIR Health, Optum/Ingenix, or proprietary payer databases, and represent the distribution of submitted charges at a specified percentile in a defined market; the source and percentile must be identified for the benchmark to be evaluable [16,17]. Payer-specific fee schedules represent a given payer’s contractually negotiated or administratively set rates for specific services and are not representative of other payers’ rates or market rates generally. Billed charges represent the provider’s list price and are not a measure of payment reasonableness, market rates, or contracted allowances. Allowed amounts represent what a payer has determined is payable for a service, irrespective of what was billed or what other payers allow. Paid amounts represent actual disbursements after application of patient cost-sharing and may substantially differ from allowed amounts. Contracted rates represent individually negotiated in-network allowances and are not representative of out-of-network market rates. Adjudicated outcomes reflect the result of a specific payment dispute and are not generalizable benchmarks.

An opinion that treats any of these constructs as interchangeable with another without explanation commits a benchmark fitness failure for purposes of Domain 4 scoring. This applies in both directions of substitution: treating a payment amount as a charge reasonableness standard without justification, and treating a charge benchmark as a payment standard without justification. The regulatory framework governing QPA calculation methodology has been subject to ongoing legal and regulatory activity since the NSA’s enactment; BORS100 does not depend on any specific QPA methodology remaining in effect. The framework asks whether the expert explains what the benchmark measures, what it excludes, and why it applies to the specific dispute: a transparency requirement that applies regardless of which version of any rule is in effect.

High-reliability features include an explanation of what each cited benchmark measures, what it excludes, why it was selected over available alternatives, and how its structural characteristics affect the conclusion in this specific dispute. Low-reliability features include benchmark recitation without explanation, benchmark substitution without justification in either direction, and failure to discuss alternative benchmarks. This domain does not privilege any specific benchmark; it evaluates whether the benchmark’s relationship to the dispute is demonstrated, not assumed.

Domain 5: Case-Specific Clinical or Procedural Linkage (15 Points)

This domain evaluates whether clinical or procedural facts are considered when they are relevant to the opinion. Relevance should be assessed based on the nature of the opinion: coding opinions addressing purely administrative questions may require less clinical record engagement than charge reasonableness opinions for complex surgical services.

High-reliability features include operative record review when surgical complexity is at issue, acknowledgment of emergency status, acuity, modifiers, and relevant procedural facts, and explicit deferral or referral to a clinician for clinical opinions outside the billing reviewer’s expertise. Low-reliability features include substituting the CPT descriptor for clinical record review in complex surgical cases, absence of emergency or complexity acknowledgment in emergency reconstructive or trauma cases, and implied characterization of operative complexity based on billing codes alone.

When the opinion is limited to purely administrative coding questions, CPT code selection from a defined descriptor set, modifier application to documented codes, or billing-form compliance with a stated standard, clinical record engagement is generally not required. Domain 5 should be scored conservatively to reflect that limitation rather than penalizing the opinion for not engaging clinical records that it was not asked to review. Reviewers should not treat the absence of clinical record engagement as a Domain 5 failure when the opinion’s stated scope does not require it.

Where clinical records are relevant to the opinion but were not made available to the reviewing expert, the rater should note this as a limiting condition rather than assigning a low score solely because of record unavailability. Domain 5 evaluates whether available relevant records were engaged, not whether a party succeeded in obtaining them.

Domain 6: Transparency and Auditability (10 Points)

This domain evaluates whether another reviewer can audit the analysis from the disclosed materials without access to the original witness. Domain 3 evaluates whether a method exists and is described; Domain 6 evaluates whether that method’s inputs, calculations, and data trail are independently verifiable from the disclosed materials. An opinion may have a described method that is not auditable (Domain 3 high, Domain 6 low) or a partially described method whose calculation trail is fully traceable (Domain 3 moderate, Domain 6 high); these distinctions matter for scoring consistency.

High-reliability features include disclosed raw benchmark values, step-by-step calculations traceable from inputs through adjustments to conclusion, version and date identification for all data sources, and explicit acknowledgment of missing data or excluded records. Low-reliability features include no traceable calculation path, undisclosed benchmark versions, missing data paths, and arithmetic conclusions without supporting inputs.

Domain 7: Bias and Independence Disclosure (10 Points)

This domain evaluates whether financial, retention, and role-based incentives are disclosed at a level sufficient to assess independence. Disclosure requirements apply symmetrically across all party positions and expert backgrounds: a provider-retained expert with an ongoing provider organization relationship, a legal practice affiliation with plaintiffs, or substantial income from provider-side engagements is subject to the same disclosure expectations as a payer-retained consultant with a concentrated payer client base, a defense-side legal affiliate, or a lien resolution service. Domain 7 does not penalize concentration per se; it evaluates whether the relevant financial and retention relationships are disclosed at a level sufficient for the adjudicator to assess independence, regardless of which side of the dispute the expert was retained to support.

High-reliability features include retention history, income source distribution, prior testimony disclosure where applicable, and disclosure of relevant financial relationships with parties or organizations. Low-reliability features include no disclosure of any kind, one-sided retention history that is undisclosed rather than disclosed, and incomplete conflict statements. This domain evaluates transparency of disclosure; it does not determine whether a disclosed relationship constitutes a disqualifying bias, which remains a judgment for the applicable adjudicator or reviewer.

Proposed 100-point rubric

Table 1 summarizes the proposed domains and point allocations. The rubric requires demonstrated evidence, not claimed consideration. A high BORS100 score does not prove that an opinion is correct; it demonstrates that the opinion’s reliability can be evaluated from the disclosed materials. A low score does not prove dishonesty, advocacy, or error; it identifies documentation and methodological gaps that limit the evaluability of the opinion.

Interpretation of score ranges

Table 2 provides conceptually proposed score profile categories for future validation. These are not validated thresholds; they are conservative, conceptually derived categories for pilot use. BORS100 should be reported as both a total score and a domain profile; a bare total score creates false precision and should not be used in isolation.

**Table 2:** Conceptually proposed score-profile categories for future validation. All score categories in the table are conceptually proposed and require empirical calibration before use as institutional standards or formal decision criteria.

Score range	Interpretation	Caution	Recommended use
85–100	Highly structured and well-documented opinion profile	Not proof of correctness; high score does not establish that the conclusion is right	May support reviewer confidence when consistent with applicable professional and legal standards; domain profile and narrative required. Pilot-use descriptive category only
70–84	Generally structured opinion profile with identifiable limitations	May require supplementation or clarification in deficient domains	Use with awareness of limitations; examine deficient domains before relying heavily. Pilot-use descriptive category only
55–69	Mixed evaluability profile requiring careful review	Meaningful methodological or documentation gaps likely remain	Use primarily to identify supplementation needs; limit reliance. Pilot-use descriptive category only
40–54	Substantial documentation or methodology limitations	Opinion may be more descriptive than analytical	Limit or qualify reliance unless gaps are corrected. Pilot-use descriptive category only
Below 40	Severe evaluability limitations	Score does not prove bad faith or substantive incorrectness	Requires major supplementation before the opinion profile can be meaningfully evaluated. Pilot-use descriptive category only

In practice, a high score should be interpreted only as evidence that the opinion is more evaluable from disclosed materials, not that the expert is more credible or that the conclusion is correct. A low score should be interpreted only as evidence that missing documentation, unclear methods, poor benchmark explanation, inadequate case linkage, or insufficient disclosure limit independent review. It should not be used by itself as a proxy for credibility, admissibility, evidentiary weight, or payment reasonableness.

The preferred reporting format is: (1) total pilot-use score; (2) domain subscores for all seven domains; (3) identification of critical missing materials and their effect on scoring; (4) identification of domains limited by unavailable materials; (5) narrative explanation of what the score means and what it does not mean; and (6) a misuse safeguard statement confirming that the score is not being used as a standalone admissibility, payment, or credibility determination.

Table 3 maps common expert opinion failure modes to the BORS100 domains to show how the framework operationalizes recurring reliability limitations.

**Table 3:** Common expert-opinion failure modes mapped to BORS100 domains. BORS100: Billing Opinion Reliability Score 100

Failure mode	Relevant domain	Why it matters	How BORS100 captures it
Undefined opinion category	Opinion scope and qualification fit	Different questions require different expertise, evidence, and standards; blended opinions cannot be evaluated against the appropriate standard for any of them	Requires explicit statement of what is and is not being opined on; scores scope definition separately from qualifications
Credential-to-opinion mismatch	Opinion scope and qualification fit	General billing experience may not support clinical complexity, medical necessity, or health economic opinions	Scores qualification fit separately from general credentials; identifies the specific opinion for which qualifications are claimed
Benchmark recitation without explanation	Benchmark literacy and fitness-for-purpose	Benchmarks measure different constructs, have structural limits, and may not apply to specific dispute types	Requires explanation of what each benchmark measures, what it excludes, why it applies, and how it affects the conclusion for this dispute
Benchmark substitution without justification (either direction)	Benchmark literacy and fitness-for-purpose	Treating a payment standard as charge reasonableness evidence, or a charge benchmark as a payment standard, without justification misrepresents what the benchmark measures	Requires disclosure of what the selected benchmark measures and why it applies; penalizes unjustified substitution in both directions
Experience substituted for method	Methodology and reproducibility	Experience explains familiarity but cannot be audited unless translated into reproducible analytical steps	Awards credit for written methods and traceable reasoning; does not credit experience alone regardless of credential level
CPT code used as clinical-record substitute in complex case	Case-specific clinical or procedural linkage	CPT descriptors do not capture acuity, tissue condition, emergency status, or operative complexity	Requires clinical or procedural record review when relevant; distinguishes descriptor review from operative record review; scales to opinion type
No calculation trail for numerical conclusions	Transparency and auditability	Numerical conclusions cannot be checked, challenged, or reproduced without inputs and calculation steps	Requires raw values, source versions, assumptions, and step-by-step calculations traceable to conclusion
Undisclosed retention pattern or financial relationship	Bias and independence disclosure	Potential incentives cannot be assessed without disclosure; applies to all experts regardless of party or background	Requires financial, retention, income source, and prior testimony disclosures when applicable; symmetrically applied to all parties
Selective record review	Source and documentation support	An expert who reviews only records favorable to the conclusion and omits unfavorable records without explanation has not engaged the full evidentiary record	Requires explicit identification of excluded records and the basis for exclusion; flags record selection appearing limited to favorable materials without explanation

The Appendices provide the editable BORS100 structured review worksheet for pilot-use reporting, including identification fields, materials reviewed tables, domain scoring, narrative interpretation, misuse safeguards, and reviewer acknowledgment sections.

## Discussion

Use cases and boundaries

Potential use cases for BORS100 include internal quality review of expert reports, attorney preparation for evaluating retained or opposing opinions, structured review in payer-provider dispute settings, support for arbitration submissions, administrative review processes, workers’ compensation proceedings, lien dispute contexts, policy-adjacent reimbursement discussions, and academic study of expert opinion quality variation. In each setting, BORS100 should be framed as a structured review aid rather than a binding rule, and reviewers should exercise professional judgment in applying domain profiles to specific contexts.

The framework functions as a preparation and quality improvement tool as well as an evaluation tool. Retained experts and the attorneys who work with them can use the domain structure to identify which elements should be documented before an opinion is finalized. A physician expert preparing a charge reasonableness opinion can use BORS100 to confirm that the opinion defines its scope, lists records reviewed, explains benchmark selection, shows calculations, addresses clinical complexity when relevant, and discloses retention relationships. Using the domain structure as a preparation checklist does not depend on validation of the numerical score, provided the framework is accurately described as a checklist and not as a validated scoring instrument.

NSA/IDR context

In the NSA/IDR context, expert opinions are typically submitted in writing as part of offer packages rather than as live testimony, and certified IDR entities consider multiple statutory factors in selecting between the parties’ final offers. BORS100 may be used to evaluate the transparency and methodological completeness of expert opinion support included in IDR submissions, but it does not determine which statutory factor should be given greatest weight, does not constitute a position on the appropriate benchmark hierarchy in IDR, and does not replace the certified IDR entity’s statutory decision-making authority. Parties referencing BORS100 in IDR submissions should characterize it as a structured review framework, not as an independent expert methodology or an evidentiary standard.

Workers’ compensation context

In workers’ compensation contexts, billing and reimbursement opinions may address fee schedule compliance, medical necessity, coding accuracy for work-related injuries, and the relationship between documented functional limitations and billed services. Workers’ compensation fee schedules are state-specific and differ structurally from Medicare fee schedules, commercial payer rates, and usual-and-customary charge data. An opinion that applies a non-workers’ compensation benchmark to a workers’ compensation fee schedule dispute without explaining the crosswalk commits a benchmark fitness failure under Domain 4. BORS100 applies in workers’ compensation settings with the same domain structure, with Domain 5 clinical linkage scaling applied to the specific opinion type.

Because workers’ compensation systems vary substantially by jurisdiction, BORS100 should not assume that any single fee schedule, modifier rule, dispute process, or benchmark hierarchy applies across states. Reviewers should identify the governing jurisdiction, applicable fee schedule or payment rule, date of service, and any state-specific documentation requirements before scoring benchmark fitness or methodology. Jurisdiction-specific adaptation may be required for formal use.

Lien dispute and collections context

In lien dispute and collections contexts, reimbursement opinions may address the reasonableness of billed charges, the relationship between billed charges and applicable payment benchmarks, and the clinical or procedural support for the services rendered. Opinions in these settings that provide numerical conclusions, such as a percentage reduction from billed charges, without disclosing the benchmark source, version, percentile, selection rationale, and calculation path, present a Domain 3, Domain 4, and Domain 6 failure profile simultaneously. BORS100 may be used to organize a structured review of lien dispute opinions using the same domain criteria, with the same symmetry requirements, as in litigation and IDR contexts.

Courtroom and Daubert contexts

A court conducting a Daubert reliability hearing may find the framework’s domain structure useful as an organizational checklist for identifying questions to ask: whether the expert defined the opinion scope, whether the qualifications match that scope, whether sufficient facts and data were identified, whether a visible method was applied, and whether the method was applied to the specific case facts. BORS100 does not answer those questions; it organizes them. A court that uses BORS100 as an organizational reference should not treat it as a legally operative standard or permit its scores to be presented as an independent admissibility determination.

Boundaries of application

Parties applying BORS100 to evaluate opposing opinions should apply the same framework symmetrically to their own retained opinions. The framework does not provide a selective review tool; its value depends on consistent application across all opinions in the dispute, regardless of which party offered them.

When BORS100 scores are disputed between parties, resolution should proceed by reference to the disclosed record rather than argument about the reviewer’s interpretive judgment. If a score cannot be traced to a specific feature of the disclosed materials, the score should be revised or the limitation acknowledged in the narrative explanation. Score disputes that cannot be resolved from the disclosed record are often the product of genuinely missing materials; the appropriate remedy is to characterize the score as limited by unavailable materials and request supplementation.

BORS100 should not be used to label an expert witness as dishonest or acting in bad faith, to determine legal admissibility by formula, to replace judicial or arbitral discretion, to substitute for applicable evidentiary rules, or to resolve the underlying billing dispute. It evaluates the documented reliability of the opinion process, not the substantive correctness of the conclusion.

The framework is designed not to suppress legitimate expert disagreement. Two well-supported opinions, both scoring in the high-reliability band, may reach different conclusions because they apply different benchmark hierarchies, different assumptions about clinical relevance, or different interpretations of applicable standards. A high BORS100 score does not privilege one conclusion over another. The framework evaluates whether the reasoning is disclosed and methodologically coherent; it does not determine which well-reasoned conclusion is more persuasive.

Legal and administrative relevance

Billing and reimbursement expert opinions frequently enter legal, administrative, and arbitral settings governed by specific evidentiary or procedural rules. BORS100 may help organize questions relevant to those settings, but does not create, modify, or replace any evidentiary rule, statutory payment standard, arbitration procedure, or administrative requirement.

BORS100 should not be presented as an expert methodology that independently satisfies Federal Rule of Evidence 702, Daubert, Kumho Tire, or any state law equivalent. It is a proposed organizing framework for reviewing disclosed opinion features, not a validated evidentiary method. Any reference to BORS100 in a legal, arbitral, or administrative setting should be accompanied by the framework’s stated limitations and should not be presented as an independent rule of decision or an authority that determines admissibility, credibility, or evidentiary weight.

Its domains identify reliability features that adjudicators, reviewers, and parties may already consider: defined scope, sufficient source support, transparent reasoning, benchmark fitness, case-specific application, auditability, and disclosure. The framework organizes those features; it does not independently determine admissibility, credibility, payment reasonableness, or evidentiary weight.

For any dispute in which an administrative, statutory, commercial, or payer-derived benchmark is cited, the framework asks whether the expert explains what the benchmark measures, what it excludes, why it applies to the specific dispute, and how it affects the conclusion. BORS100 does not take a position on the appropriate statutory role of the QPA or any other administrative reference [5,14,15].

Limitations

BORS100 is not a finished instrument. It is a structured proposal. Proposals can be refined, validated, and improved; this one should be. The limitations described here are not arguments for abandoning structured review; they are arguments for correct characterization. Domain profiles, missing element identification, and narrative explanation are the framework’s current strengths. Total score comparisons, score band classifications, and institutional thresholds require empirical calibration before they can be treated as validated measures.

BORS100 is a proposed conceptual framework. It is not a validated psychometric instrument, legal test, clinical guideline, payment standard, or regulatory rule. Its present defensible use is as a conceptual framework, structured checklist, and pilot-use scoring aid for organizing review of disclosed opinion materials. The seven domains, point allocation, and score interpretation bands reflect conceptual judgments about expert opinion reliability derived from published reliability principles and structured professional judgment literature. They require empirical testing before adoption as a formal scoring instrument or institutional standard.

The 100-point additive scale may create an impression of precision greater than the current evidence supports. This limitation should be addressed by reporting domain profiles, applying score bands conservatively, and providing narrative explanations rather than relying on exact total scores. A difference of two or three points in total score should not be treated as meaningful without examining the domain profile that produced the difference. The scale is appropriately understood as ordinal and descriptive during pilot use.

Domain weights are not empirically derived. Although the rationale for differential weighting is articulated in the Methodology section, the current allocation reflects the authors’ conceptual judgment and has not been empirically tested. Future validation should examine whether the current weights produce meaningfully different rankings compared with equal-weight alternatives.

Inter-rater reliability has not been established. Without demonstrated agreement among trained raters, the framework’s total scores and domain profiles may vary substantially depending on who applies them. This limitation makes it inappropriate to use BORS100 scores as formal adjudicative criteria or institutional thresholds until inter-rater reliability data are available.

No gold standard for correct billing opinion quality exists. Available outcomes, judicial limitations on opinion weight, arbitral requests for supplementation, settlements suggesting contested quality, and independent expert quality ratings, are all imperfect proxies. This is a constraint shared by all structured professional judgment frameworks and does not preclude using BORS100 as a structured review aid; it means only that empirical validation of score band calibration will use imperfect outcome proxies rather than an objective truth criterion. Validation outcomes can test whether BORS100 tracks review consequences, not whether it measures truth.

The framework may require domain-specific modification for different opinion types. Domain 5 is directly relevant to complex surgical charge opinions but may require recalibration for purely administrative coding questions; the scaling provision in Domain 5 addresses but does not fully resolve this variation.

BORS100 may be misused if applied mechanically, directionally, or as a litigation label. The framework should not function as a vehicle for suppressing legitimate expert testimony, dismissing professionally reasonable dissenting opinions, or substituting a scoring exercise for substantive engagement with the expert’s conclusions.

Future validation pathways

**Figure 2:**
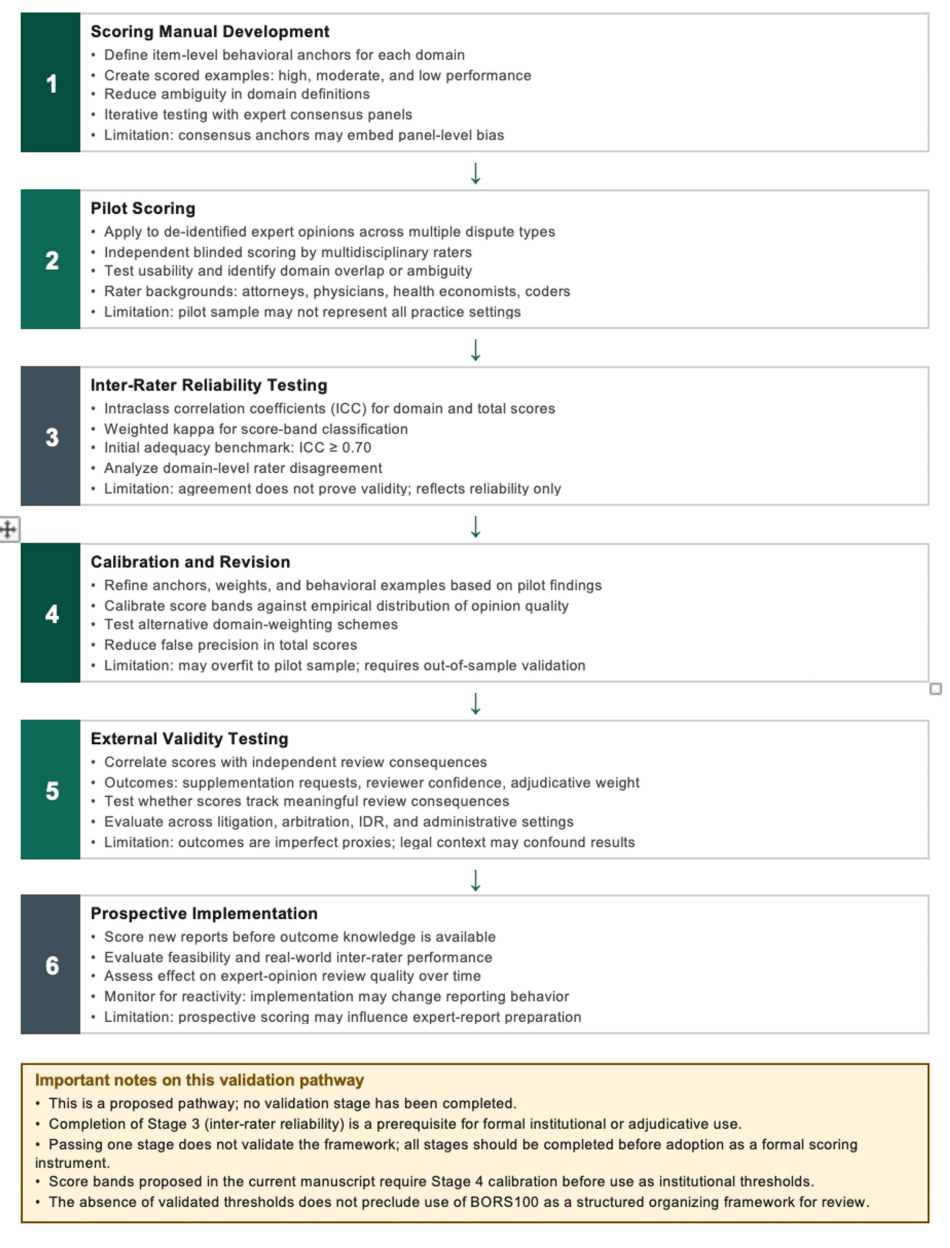
Proposed BORS100 validation pathway. Proposed BORS100 validation pathway. Framework development proceeds from scoring manual creation through pilot scoring, inter-rater reliability testing, calibration and revision, external validity testing, and prospective implementation. Inter-rater reliability should be assessed using ICCs for domain and total scores and weighted kappa for score-band classification, with ICC greater than or equal to 0.70 used as an initial adequacy benchmark. Each stage should evaluate whether the framework improves transparency, reproducibility, and consistency in expert opinion review while acknowledging that reliability does not prove validity and that external outcomes are imperfect proxies for opinion quality. BORS100: Billing Opinion Reliability Score 100; ICC: intraclass correlation coefficient

Table 4 presents a proposed six-stage validation program. The stages are sequential but not necessarily discrete; pilot findings should inform manual refinement before broader inter-rater testing. Figure 2 summarizes the validation pathway and emphasizes that no validation stage has been completed at the time of this publication.

**Table 4:** Future validation strategy. BORS100: Billing Opinion Reliability Score 100; ICC: intraclass correlation coefficient

Validation step	Data source	Method	Purpose	Limitation
Scoring manual development	Representative opinion excerpts; expert consensus panels	Define behavioral anchors for high, moderate, and low scores per domain	Reduce ambiguity; improve rater consistency	Consensus-based anchors may embed expert-panel bias
Pilot scoring	De-identified opinions from multiple dispute types; sample size to be justified by rater count and expected ICC	Independent blinded scoring by multidisciplinary raters after standardized training	Test usability; identify domain overlap or ambiguity	Sample may not represent all practice settings
Inter-rater reliability testing	Repeated scoring sets with the same opinions scored by multiple raters	ICC for total and domain scores; pre-specified thresholds (≥0.70 preliminary, ≥0.80 for high-stakes); weighted kappa for band classification	Assess whether different raters apply BORS100 consistently	Agreement does not prove validity; thresholds must be pre-specified
Calibration and revision	Rater comments; disagreement analysis; domain-specific agreement data	Refine language, weights, score bands, and behavioral anchors	Improve clarity; reduce false precision	May overfit to pilot sample; requires out-of-sample validation
External validity testing	Adjudicative outcomes; supplementation requests; independent quality ratings	Correlate BORS100 scores with independent outcomes	Assess whether scores track meaningful review consequences	Outcomes are imperfect proxies; no gold standard for opinion correctness
Prospective implementation	New reports scored before outcome knowledge	Evaluate the feasibility, inter-rater performance in practice, and the effect on review quality	Test real-world utility	Implementation may change reporting behavior; reactivity effects likely

Rater selection should include multidisciplinary participants, i.e., attorneys, physicians, health economists, coders, and reimbursement analysts, to test whether the framework produces consistent results across rater backgrounds. A pilot cohort of 30-50 de-identified expert opinions is the proposed starting target; this figure is a practical starting point rather than a statistically derived sample size, and the appropriate sample will depend on the number of raters, expected intraclass correlation coefficients (ICCs), and the precision of the resulting confidence interval.

Inter-rater reliability should be evaluated using ICCs for domain and total scores and weighted kappa for band classification. An ICC ≥0.70 is commonly cited as a preliminary benchmark for acceptable inter-rater reliability in structured rating contexts [19,20]; thresholds of 0.80 or higher are appropriate for high-stakes applications. ICC targets should be pre-specified before inter-rater testing to prevent post-hoc threshold selection.

Future validation should include multidisciplinary collaboration across professional communities, i.e., attorneys, providers, payers, coders, health economists, and arbitrators, to test whether the framework’s domain structure and weighting are perceived as symmetrical and methodologically defensible across perspectives. The authors invite such collaboration.

## Conclusions

Expert opinions that cannot be evaluated from their disclosed materials may impose practical costs on the adjudicative, administrative, and clinical systems that rely on them. Billing and reimbursement disputes require more than credentials, benchmark citations, or conclusory experience statements; they require visible reasoning that can be reviewed, challenged, reproduced, or supplemented. BORS100 proposes a structured basis for distinguishing evaluable from non-evaluable opinions. It does not resolve disputes, determine payment, establish legal admissibility, or replace professional judgment. It organizes the features that make an opinion reviewable: defined scope, qualification fit, source support, disclosed method, benchmark fitness analysis, case-specific linkage when relevant, auditability, and independence disclosure. Future research should test inter-rater reliability, refine scoring anchors, calibrate score bands across dispute types, and evaluate external validity using imperfect but transparent outcome proxies. The authors invite multidisciplinary collaboration across legal, provider, payer, health economic, and coding communities to advance the validation program. Until validation is completed, the framework’s organizational value, i.e., visible reasoning, disclosed method, benchmark fitness analysis, case-specific linkage, and auditability, is available to anyone who chooses to apply it.

## Appendices

**Table 5:** Section 1A. Review identification.

Field	Response
Matter/case/review identifier:	
Date of review:	
Reviewer name/role:	
Reviewer domain qualifications:	
Expert report reviewed (title, date):	
Expert name/credential/role:	

**Table 6:** Section 1B. Opinion type.

Opinion type	Check/Response
Coding accuracy	☐
Documentation sufficiency	☐
Billed-charge reasonableness	☐
Payment reasonableness	☐
Benchmark interpretation	☐
Medical necessity	☐
Clinical or procedural complexity	☐
Payer-policy compliance	☐
Workers’ compensation fee-schedule compliance	☐
Lien-dispute charge reasonableness	☐
Other:	☐

**Table 7:** Section 2A. Materials reviewed.

Material category	Identified (title/date/version)	Reviewed (yes/no/partial/unavailable)
Expert report		
Curriculum vitae/Qualification materials		
Records-reviewed list provided by the expert		
Medical records/Operative reports		
Billing records/Claim forms/EOBs		
Benchmark source documents (with version, percentile, and date)		
Workpapers/Calculations		
Disclosure/Conflict-of-interest materials		
Prior testimony list (if applicable)		
Other materials:		

**Table 8:** Section 2B. Unavailable materials.

Item	Response
Materials requested but not available:	
Limitations caused by unavailable materials:	

**Table 9:** Section 3. Domain scoring table.

Domain	Maximum	Pilot-use points	Evidence supporting score	Missing/Limiting information	Reviewer notes
1. Opinion scope and qualification fit	15				
2. Source and documentation support	15				
3. Methodology and reproducibility	20				
4. Benchmark literacy and fitness-for-purpose	15				
5. Case-specific clinical or procedural linkage	15				
6. Transparency and auditability	10				
7. Bias and independence disclosure	10				
Total	100	__/100			

**Table 10:** Section 4A. Total pilot-use score and domain profile.

Summary item	Response
Reviewer-assigned pilot-use total:	
Highest-strength domains:	
Lowest-strength domains:	
Critical missing elements:	
Domains limited by unavailable materials:	

**Table 11:** Section 4B. Score interpretation band.

Check	Score range	Interpretation
☐	85–100	Highly structured and well-documented opinion profile; domain profile review and narrative required before reliance
☐	70–84	Generally structured opinion profile with identifiable limitations; examine deficient domains before relying heavily
☐	55–69	Mixed evaluability profile requiring careful review; identify supplementation needs
☐	40–54	Substantial documentation or methodology limitations; identify supplementation needs before reliance
☐	Below 40	Severe evaluability limitations; requires major supplementation before meaningful evaluation

**Table 12:** Section 4C. Pilot-use caution.

Pilot-use caution
BORS100 is not empirically validated. Score bands are conceptually proposed and require empirical calibration. A high score does not prove correctness, and a low score does not prove bad faith. Report total score with domain subscores, narrative explanation, and limiting conditions

**Table 13:** Section 5. Narrative interpretation.

Narrative item	Response
Summary of reliability profile:	
Key methodological strengths:	
Key methodological limitations:	
Missing materials affecting interpretation:	
Scope limitations or out-of-scope opinion elements:	
Benchmark fitness assessment (Domain 4 narrative):	
Recommended supplementation (if any):	
Final narrative interpretation:	

**Table 14:** Section 6. Misuse safeguards.

Check	Misuse safeguard
☐	The score is reported with domain subscores, not as a standalone total
☐	The score is accompanied by a written narrative explanation
☐	Missing or unavailable materials are specifically identified, and their effect on scoring is described
☐	Scoring limitations are disclosed, including limitations caused by material unavailability
☐	The framework is not being used as a standalone legal admissibility test
☐	The framework is not being used as a standalone payment-determination tool
☐	The framework is not being used to label the expert as dishonest, biased, or acting in bad faith
☐	The framework is applied symmetrically to all retained opinions in the matter, regardless of which party offered them
☐	Score bands are identified as conceptually proposed, not validated thresholds
☐	The reviewer acknowledges that BORS100 has not yet been validated, and scores may vary depending on the reviewer and materials
☐	The reviewer has sufficient domain knowledge to evaluate the disclosed materials against the scoring criteria
☐	The framework is not being presented as satisfying Federal Rule of Evidence 702, Daubert, Kumho Tire, or any state-law equivalent
☐	If used for self-review or report preparation, that use is identified as self-review and is not presented as independent scoring
☐	When BORS100 scores are disputed, the reviewer is prepared to resolve the dispute by reference to the disclosed record

**Table 15:** Section 7A. Reviewer acknowledgment fields.

Field	Response
Reviewer name:	
Reviewer role/title:	
Organizational affiliation:	
Date of review:	
Reviewer initials (if used):	

**Table 16:** Section 7B. Reviewer acknowledgment statement.

Reviewer acknowledgment statement
I understand that BORS100 is a proposed conceptual structured review framework that has not yet been empirically validated. This worksheet reflects my structured review of disclosed materials using the proposed BORS100 domains and pilot-use scoring constraints. The score and domain profile do not independently determine truth, admissibility, credibility, payment obligation, adjudicative outcome, or substantive correctness. Scores are presented with domain subscores, narrative explanation, and identified limitations. I have applied the framework symmetrically and would apply the same standard to all opinions in this matter, regardless of which party offered them. I am not presenting BORS100 as a validated instrument, legal admissibility standard, or payment-determination tool
